# CCL5-Secreting Virtual Memory CD8+ T Cells Inversely Associate With Viral Reservoir Size in HIV‐1−Infected Individuals on Antiretroviral Therapy

**DOI:** 10.3389/fimmu.2022.897569

**Published:** 2022-05-26

**Authors:** Wei Hu, Yan-Jun Li, Cheng Zhen, You-Yuan Wang, Hui-Huang Huang, Jun Zou, Yan-Qing Zheng, Gui-Chan Huang, Si-Run Meng, Jie-Hua Jin, Jing Li, Ming-Ju Zhou, Yu-Long Fu, Peng Zhang, Xiao-Yu Li, Tao Yang, Xiu-Wen Wang, Xiu-Han Yang, Jin-Wen Song, Xing Fan, Yan-Mei Jiao, Ruo-Nan Xu, Ji-Yuan Zhang, Chun-Bao Zhou, Jin-Hong Yuan, Lei Huang, Ya-Qin Qin, Feng-Yao Wu, Ming Shi, Fu-Sheng Wang, Chao Zhang

**Affiliations:** ^1^ Medical School of Chinese People’s Liberation Army (PLA), Beijing, China; ^2^ Department of Infectious Diseases, The Fifth Medical Center of Chinese PLA General Hospital, National Clinical Research Center for Infectious Diseases, Beijing, China; ^3^ Guangxi Acquired Immune Deficiency Syndrome (AIDS) Clinical Treatment Centre, The Fourth People’s Hospital of Nanning, Nanning, China

**Keywords:** HIV-1 reservoir, HIV-1 cure, CD8+ T cells, virtual memory CD8+ T cells, CCL5

## Abstract

Recent studies highlighted that CD8+ T cells are necessary for restraining reservoir in HIV-1-infected individuals who undergo antiretroviral therapy (ART), whereas the underlying cellular and molecular mechanisms remain largely unknown. Here, we enrolled 60 virologically suppressed HIV-1-infected individuals, to assess the correlations of the effector molecules and phenotypic subsets of CD8+ T cells with HIV-1 DNA and cell-associated unspliced RNA (CA usRNA). We found that the levels of HIV-1 DNA and usRNA correlated positively with the percentage of CCL4+CCL5- CD8+ central memory cells (T_CM_) while negatively with CCL4-CCL5+ CD8+ terminally differentiated effector memory cells (T_EMRA_). Moreover, a virtual memory CD8+ T cell (T_VM_) subset was enriched in CCL4-CCL5+ T_EMRA_ cells and phenotypically distinctive from CCL4+ T_CM_ subset, supported by single-cell RNA-Seq data. Specifically, T_VM_ cells showed superior cytotoxicity potentially driven by T-bet and RUNX3, while CCL4+ T_CM_ subset displayed a suppressive phenotype dominated by JUNB and CREM. In viral inhibition assays, T_VM_ cells inhibited HIV-1 reactivation more effectively than non-T_VM_ CD8+ T cells, which was dependent on CCL5 secretion. Our study highlights CCL5-secreting T_VM_ cells subset as a potential determinant of HIV-1 reservoir size. This might be helpful to design CD8+ T cell-based therapeutic strategies for cure of the disease.

## Introduction

Established antiretroviral therapy (ART) efficiently suppresses HIV-1 replication and *de novo* infection arising in people living with HIV-1 (PLWH). The implementation of a “treat-all” policy has led to infection with HIV-1 becoming a manageable chronic health condition (1–3). However, lifelong ART is mandatory to maintain viral suppression because latent HIV-1 reservoirs are major obstacles to curing HIV-1 (4).

Several clinical parameters are associated with the size of HIV-1 reservoir, such as pre-ART CD4 cell count (5), the CD4/CD8 ratio (5) and HIV-1 viral load (6, 7), residual viral replication during ART (6), and the duration of ART (6, 7). Moreover, the immune system, especially CD8+ T cells, plays an essential role in constraining HIV-1 reservoirs and limiting HIV-1 rebound during ART (8, 9). Defining evidence has been generated from SIV-infected macaques with viral suppression under ART (10). Plasma viremia develops in all animals when CD8+ T cells are depleted, and this can be controlled by repopulation with CD8+ T cells (10, 11). In PLWH who receive early ART, a preserved CD8+ T-cell effector function is associated with a smaller reservoir (12, 13). Thus, CD8+ T cells are important in limiting the HIV-1 reservoir in PLWH and might be harnessed to induce therapeutic antiviral effects (14). However, the exact functional subset and mechanism of action are not well understood.

The polyfunctional anti-HIV-1 effects exerted by CD8+ T cells are characterized by the production of cytotoxic granules such as granzyme B (GZMB) and perforin (PRF), the secretion of effector cytokines such as interferon-γ (IFNγ), tumor necrosis factor alpha (TNFα) and interleukin (IL)-2 (12, 13), as well as production of β chemokines such as chemokine (C-C motif) ligand 3 (CCL3, also known as [aka] MIP-1α), CCL4 (aka MIP-1β) and CCL5 (aka RANTES) (15). Although those molecules are often used to evaluate the anti-HIV-1 functionality of CD8+ T cells, different cytokine-producing CD8+ T-cell subsets might be heterogeneous. For example, PLWH with poor immune reconstitution showed increased frequency of CCL4+ CD8+ T cells (16). In another Uganda women ART cohort, it was reported that the TNFα+ CD8+ T-cell frequency positively correlates with HIV-1 reservoir size (7).

In some cases, CD8+ T cells can be activated without cognate antigen stimulation (17). Such innate-like, bystander effect was also observed in HIV-1 infection. For instance, it was reported that major histocompatibility complex (MHC) matching is not necessary for CD8+ T cells to kill HIV-1-infected cells during primary infection (18, 19). Furthermore, bystander CD8+ T cells play an important role in maintaining the suppression of viremia in individuals undergoing ART (18, 20–25). Of which, we previously showed that a virtual memory T (T_VM_) cells subset, defined by CD44^hi^ CD122^hi^ CD49d^lo^ in mice and CD45RA+ killer Ig-like receptors (KIR)+ and/or CD94 natural killer group 2 member A (NKG2A)+ in humans (17, 26, 27), senses and inhibits the reactivation of HIV-1 reservoir through KIR (28). Thus, T_VM_ cells might represent a bystander CD8+ T-cell subset of interest in terms of constraining the size of HIV-1 reservoirs (29). The clinical relevance and functionality of this subset warrants further investigation.

The present study aimed to determine the effector mechanisms of CD8+ T cells, particularly T_VM_ cells, and their potential impacts on HIV-1 reservoir. We found CCL4-CCL5+ terminally differentiated effector memory cells (T_EMRA_) negatively correlated with HIV-1 reservoir. T_VM_ cells were enriched in CCL4-CCL5+ T_EMRA_ cells and efficiently suppressed HIV-1 *in vitro* in a CCL5 dependent manner. Our findings highlight the role of CCL5-secreting T_VM_ cells in constraining HIV-1 reservoirs and the potential of utilizing this mechanism to shrink HIV-1 reservoirs in future studies.

## Materials and Methods

### Study Participants

We enrolled PLWH from the Fourth People’s Hospital of Nanning, Nanning, China. The inclusion criteria comprised: age 18–65 years, confirmed HIV-1 positive, successful ART for > 2 years, undetectable blood HIV-1 RNA < 20 copies/mL (below the limits of detection, undetectable, ND) for at least 6 to 12 months, and a CD4 cell count > 250/μL within 7 days of clinical sample collection. Key exclusion criteria comprised co-infection with hepatitis B or C viruses, pregnancy, illicit intravenous drug use, and common comorbidities, including seizure disorder, and liver and kidney-related diseases. **Table 1** summarizes the characteristics of the participants. Individuals included in *ex vivo* functional assays were additionally recruited virologically suppressed HIV-1−infected individuals with the same inclusion criteria.

**Table 1:** Characteristics of study population (n = 60).

Characteristic	Value
Age (y)	46 (26–56)
Gender (female/male)	22/38
Pre-ART	
CD4 cell count (cells/μL)	185.5 (5–934)
CD8 cell count (cells/μL)	591.5 (50–2650)
CD4/CD8 ratio	0.23 (0.01–1.23)
Time on ART (y)	6.6 (2.5–14.9)
ART regimens−no.	
2 NRTIs + 1 NNRTIs	51 (85%)
2 NRTIs + 1 PIs	8 (13%)
1 NRTIs + 1 PIs	1 (2%)
At enrollment	
Plasma HIV RNA	ND
CD4 cell count (cells/μL)	504.5 (283–1378)
CD8 cell count (cells/μL)	743 (377–1846)
CD4/CD8 ratio	0.70 (0.24–1.87)

Data are shown as medians with ranges (min to max) or no. with percentages. ART, Antiretroviral therapy; ND, undetectable; NRTIs, nucleoside reverse transcriptase inhibitors; NNRTIs, non-nucleoside reverse transcriptase inhibitors; PIs, protease inhibitors.

### Flow Cytometry

Peripheral blood samples were collected upon enrollment, and peripheral blood mononuclear cells (PBMCs) were isolated using Ficoll-Hypaque density gradient centrifugation. Sorted PBMCs were cryopreserved in liquid nitrogen. Before phenotypic staining (detection of GZMB, GNLY and PRF) or functional stimulation (detection of CCL3, CCL4, CCL5, IL-2, IFNγ and TNFα), cryopreserved PBMCs were thawed and incubated in RPMI 1640 containing 10% fetal bovine serum (FBS) for 2 h at 37°C and 5% CO_2_. Then cells were stained with monoclonal antibody (mAb) for surface markers at 4°C for 30 min. For intracellular staining, the samples were fixed and permeabilized for intracellular staining with the indicated antibodies at 4°C for 30 min using the Foxp3/Transcription Factor Staining Buffer Set (Thermo Fisher Scientific Inc., Waltham, MA). The cells were fixed in 2% formaldehyde and examined by flow cytometry on a BD Canto II flow cytometer (BD Biosciences, San Diego, CA) within 24 h. Flow cytometry data were analyzed using FlowJo software (version 10.5.3, BD Biosciences). Aberrant events were removed using the FlowAI plugin for t-distributed stochastic neighbor embedding (t-SNE) analysis (30). The DownSample plugin was then used to randomly down-sample CD8+ T cells into 3,000 cells, which were then concatenated into a single file. This file was visualized using the t-SNE plugin with CD45RA, CD27, CCL3, CCL4, and CCL5 markers under the following conditions: Iterations 300, Perplexity 30, Eta 7% of cell number, and Theta 0.5. The subsets were then separately gated and plotted onto a t-SNE map.

The following fluorochrome-conjugated anti-human mAbs or reagents were used: APC/Fire™ 750 anti-CD3 (SK7), PerCP anti-CD8 (SK1), BV510 anti-CD45RA (HI100), PE-Cy7 anti-CD27 (O323), BV421 anti-PRF (dG9), APC anti-IL-2 (MQ1-17H12), FITC anti-IFNγ (4S.B3), and BV421 anti-TNFα (MAb11) (BioLegend, San Diego, CA); PerCP anti-CD4 (SK3), AF647 anti-GZMB (GB11), AF488 anti-GNLY (RB1), and BV421 anti-CCL5 (2D5) (BD Biosciences); PE anti-NKG2A (REA110), PE anti-KIR2D (DX27), and PE anti-KIR3DL1 (DX9) (Miltenyi Biotec, Auburn, CA); FITC anti-CCL3 (CR3M) and AF647 anti-CCL4 (FL34Z3L) (Thermo Fisher Scientific Inc.); FITC anti-Gag P24 (kc57) (Beckman Coulter Brea, CA); and Live/dead dye (Thermo Fisher Scientific Inc.).

### Functional Assays for CD8+ T Cells

We stimulated PBMCs with IL-15 (50 ng/mL) for 48 h and IL-2 (500 IU/mL), anti-CD3 (1 ng/mL), anti-CD28 (1 ng/mL), and anti-CD49d (1 ng/mL) for 6 h to detect intracellular CCL3, CCL4, and CCL5 (31), and with IL-12 (20 ng/mL), IL-15 (10 ng/mL), and IL-18 (20 ng/mL) for 48 h to detect intracellular IL-2, IFNγ and TNFα (26). GolgiPlug was added to the medium 6 h before harvest to detect intracellular cytokines. Recombinant human IL-12 and IL-18 were purchased from PeproTech (Rocky Hill, NJ). Recombinant human IL-2 and IL-15, anti-human CD3 (clone OKT3), anti-human CD28 (clone CD28.2), and anti-human CD49d (clone 9F10) antibodies were purchased from BioLegend.

### Detection of HIV-1 DNA and CA usRNA

Total cellular HIV-1 DNA and RNA were extracted using Qiagen QIAsymphony DNA Mini Kits (Qiagen, Valencia, CA) and HiPure Total RNA Plus Mini Kits (Magen, Guangzhou, China), respectively. We quantified HIV-1 DNA and CA usRNA using fluorescence-based real-time SUPBIO HIV-1 Quantitative Detection Kits (SUPBIO, Guangzhou, China). The quantitation range was 5–10 ×10^6^ copies/10^6^ PBMCs.

### Viral Inhibition Assay

Latency-reversal CD4+ T cells were co-cultured with killer cells to analyze their suppressive capability as described with minor modifications (32–34). Briefly, CD8+ T and CD56+ NK cells were isolated using anti-CD8 and anti-CD56 microbeads (Miltenyi Biotec), respectively. CD8+ T cells were maintained in RPMI 1640 medium containing 10% FBS and 500 IU/mL IL-2 before coculture. The remaining CD8− and CD56−depleted cells were mainly CD4+ T cells with > 90% purity, and were cultured in RPMI 1640 medium containing 10% FBS, 50 ng/ml PMA, 1 μM ionomycin, and 500 IU/mL IL-2 for 48 h. Latency-reversal CD4+ T cells were cultured alone or co-cultured with CD8+ T cells at a 1:3 ratio in RPMI 1640 medium containing 10% FBS and 500 IU/mL IL-2 for 24 h. Viral inhibition was determined as a decrease in P24+ % CD4+ T cells and as % inhibition.

The inhibition abilities of T_VM_ and non-T_VM_ were compared as follows. The CD8+ T cells selected using microbeads were further sorted into T_VM_ and T_VM_−depleted (non-T_VM_ CD8+ T cells) subsets using a FACS Aria II cell sorter (BD Bioscience). The sorted subsets were separately cultured for 2 days, then co-cultured with latency-reversal CD8− and CD56−depleted cells to calculate viral suppression rates as described above. To examine the inhibition mechanisms of T_VM_ cells, 5 μg/mL CCL5 blockade antibody (αCCL5, clone 21445, R&D Systems, Minneapolis, MN) and 5 μg/mL αCCL3 (clone W16009B, BioLegend) were separately added to the co-culture system.

### scRNA-seq Analysis

The raw scRNA-seq data of CD8+ T cells purified from PBMCs of 3 ART-treated individuals (clinical characteristics were presented in **Supplementary Figure 6A**) were downloaded from the Genome Sequence Archive of the Beijing Institute of Genomics Data Center, Chinese Academy of Sciences [http://bigd.big.ac.cn/gsa-human, accession code HRA000190, uploaded by our group (35)]. The downloaded data were processed with Cell ranger (v.6.0.1) pipeline against GRCh38 human reference genome to generate gene expression matrices. Subsequently, the quality control process was performed with the Seurat package (v.4.0.5) in R (v.4.1.0). Briefly, the genes expressed in ≥ 5 cells were kept for every sample; cells that met these following criteria were retained: (1) > 500 genes; (2) ≥1000 but < 20000 unique molecular identifiers (UMIs); (3) <10% UMIs derived from the mitochondrial genome (**Supplementary Figure 6B**). Scrublet package (v.0.2.3) in Python (v.3.9.7) was applied to each dataset to remove potential doublets with the expected doublet rate of 6%. All the cells predicted as “doublets” were filtered out. The NormalizeData function in Seurat was applied to normalize the gene expression matrices, which were then processed for dimension reduction, batch effect correction and unsupervised clustering. In principal component analysis the total number of PCs was set to 50. For clustering, the dimensions of reduction were set to 1–30 and resolution was set to 0.3.

Three scRNA-seq datasets (each for one person) were assembled with Seurat package into an integrated dataset to eliminate the batch effect. In detail, anchors among the three datasets were identified with the FindIntegrationAnchors function, and then were imported into the IntegrateData function to generate a corrected and integrated Seurat object. Subsequently, uniform manifold approximation and projection (UMAP) of all cells was performed by the RunUMAP function in Seurat to reduce the dimension and visualize the clusters. All these clusters were manually annotated according to the expression of selected markers (36).

The differentially expressed genes (DEGs) were calculated with the FindMarkers function using Wilcoxon Rank Sum test and p-values were adjusted through Bonferroni correction. For gene ontology (GO) analysis and transcription factor analysis, DEGs with log_2_ fold change (log_2_FC) of more than 0.3 and adjusted p-values of less than 0.05 were selected. In volcano plots, threshold of log_2_FC was set to 0.5 and threshold for adjusted p-values was 0.05. GO analysis were carried out with ClusterProfiler package (v.4.0.5) in R.

To reconstruct the gene regulatory networks, transcriptional factor (TF) analysis and clustering were performed with SCENIC package (v.1.2.4) in R and Arboreto package (v.0.1.6) in Python (37). Briefly, the normalized and quality-controlled single-cell RNA-seq expression matrix was used as input, while both 500 bp upstream of the transcription start site (TSS) and ±10kbp around the TSS were explored for TF binding motifs. SCENIC identify potential targets for each TF based on co-expression matrix and GRNBoost2 algorithm in Arboreto package was applied to filter out unqualified genes to form co-expression modules. Potential direct-binding targets (regulons) based on DNA-motif analysis was selected and the network activity in each individual cell was calculated with AUCell. The cell-regulon activity matrix was visualized as heatmap and the gene regulatory networks was drawn with Cytoscape (v.3.9.0).

### Statistical Analysis

Data were statistically analyzed using GraphPad Prism version 8.0.1 (GraphPad Software Inc., San Diego, CA, USA). Data are shown as medians with ranges (min to max). For two group comparison, Mann Whitney tests were used for unpaired data and Wilcoxon matched-pairs signed rank tests for paired data. For three group comparison, an overall primary analysis was performed (Kruskal-Wallis tests for unpaired data and Friedman tests for paired data). If the results were statistically significant, comparisons between two group were further performed. Correlations were evaluated using nonparametric Spearman correlation tests. Values were considered statistically significant at p < 0.05. Due to current cohort size and the exploratory nature of the study, Bonferroni corrections were employed to adjust the threshold of p value in multiple comparison analysis.

## Results

### Characteristics of Study Population

**Figure 1:**
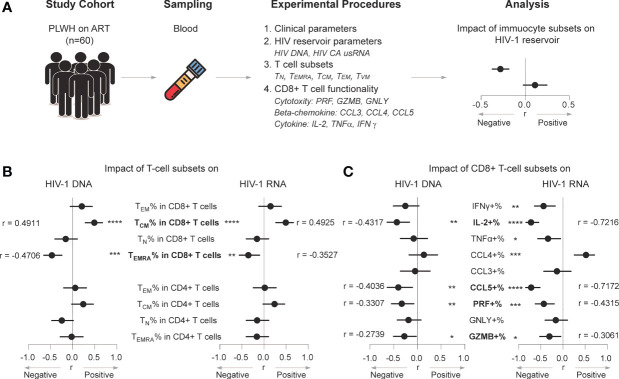
CD8+ T_EMRA_ and CCL5+ CD8+ T-cell percentages negatively correlate with the HIV-1 reservoir in ART individuals. **(A)** Scheme of study design. **(B)** Correlations between T-cell subset percentages and HIV-1 DNA or CA usRNA levels. **(C)** Correlations between functional CD8+ T percentages and HIV-1 DNA or CA usRNA levels. Correlations were evaluated using nonparametric Spearman correlation tests; Black dots denote nonparametric Spearman r, and black lines denote 95% confidence interval **(B, C)**. *P < 0.05, **P < 0.01, ***P < 0.001 and ****P < 0.0001.

The present study included 60 participants with chronic HIV-1 infection that had sustained virological suppression during ART. They had been under ART for > 2 years (median, 6.6 years). **Table 1** summarizes the characteristics of the study population. The median age was 46 years, 37% were female, the median pre-ART and present CD4 cell counts were 185.5 and 504.5/μL, respectively. **Figure 1A** shows the design of current study. The median numbers of HIV-1 DNA and HIV-1 CA usRNA copies were 217 and 468/10^6^ cells, respectively. We analyzed CD4+ and CD8+ T-cell subsets based on CD45RA and CD27 expression and effector molecule expression in CD8+ T cells and phenotypic subsets. Pre-ART CD4 cell counts and the pre-ART CD4/CD8 ratio were negatively associated with HIV-1 DNA (r = -0.2844, p = 0.0263 and r = -0.3012, p = 0.0183, respectively; **Supplementary Figure 1**). There correlations became insignificant after Bonferroni correction (p_adj set to 0.0063). Although not statistically significant, the increase in CD4 cell count tended to positively correlate with HIV-1 DNA (r = 0.2411, p = 0.0613; **Supplementary Figure 1**). ART duration was insignificantly correlated with HIV-1 DNA and immune subset parameters (**Supplementary Figures 1, 8**). Associations between the analyzed clinical parameters and HIV-1 CA usRNA were not identified.

### CD8+ T_EMRA_ Percentage Negatively Correlates With the HIV-1 Reservoir in ART Individuals

Based on CD45RA and CD27 expression, CD4+ and CD8+ T cells were classified as CD45RA-CD27- effector memory (T_EM_), CD45RA+CD27- T_EMRA_, CD45RA-CD27+ central memory (T_CM_), and CD45RA+CD27+ naïve (T_N_) populations (**Supplementary Figure 2A** shows gating strategy) (38). Statistical trends in associations were found between the CD4+ T_CM_ percentage and HIV-1 DNA (r = 0.239, p = 0.0636) and CA usRNA (r = 0.2382, p = 0.064), and between the CD4+ T_N_ percentage and HIV-1 DNA (r = -0.2450, p = 0.0570) (**Figure 1B**). No other CD4+ sub-population percentages were associated with HIV-1 DNA or CA usRNA.

Percentage of CD8+ T_CM_ positively correlated with HIV-1 DNA (r = 0.4911, p< 0.0001) and CA usRNA (r = 0.4925, p < 0.0001), whereas CD8+ T_EMRA_ percentage correlated negatively with HIV-1 DNA (r = -0.4706, p = 0.0001) and CA usRNA (r = -0.3527, p = 0.0057) (**Figure 1B** and **Supplementary Figure 3**). These correlations remained statistically significant after Bonferroni corrections (p_adj set to 0.0063). The CD8+ T_EM_ or T_N_ percentages were not associated with HIV-1 DNA or CA usRNA.

### CCL5 Expression in CD8+ T Cells Negatively Correlates With HIV-1 Reservoir Size

CD8+ T cells are important in restraining HIV-1 rebound and reservoir size (10, 28, 39). We further analyzed effector molecule expression in CD8+ T cells (**Supplementary Figure 2B** for gating strategy) and their correlations with HIV-1 DNA and CA usRNA. The percentages of CCL5+, IL-2+, PRF+, and GZMB+ CD8+ T cells were negatively associated with HIV-1 DNA (CCL5+ CD8+ T cells, r = -0.4036, p = 0.0080; IL-2+ CD8+ T cells, r = -0.4317, p = 0.0017; PRF+ CD8+ T cells, r = -0.3307, p = 0.0098; GZMB+ CD8+ T cells, r = -0.2739, p = 0.0342) and CA usRNA (CCL5+ CD8+ T cells, r = -0.7172, p < 0.0001; IL-2+ CD8+ T cells, r = -0.7216, p < 0.0001; PRF+ CD8+ T cells, r = -0.4315, p = 0.0006; GZMB+ CD8+ T cells, r = -0.3061, p = 0.0174) (**Figure 1C** and **Supplementary Figure 3**). After Bonferroni correction, there were no significant correlation between the above effector molecule expressed CD8+ T cells and HIV-1 DNA, with exception for IL-2+ CD8+ T cells, no significant correlation between GZMB+ CD8+ T cells and HIV-1 CA usRNA, but still significant correlations between IL-2+, CCL5+ and PRF+ CD8+ T cells and HIV-1 CA usRNA (p_adj set to 0.0056). In agreement with the crucial role of PRF in the killing effects of GZMB, percentages of GZMB+ polyfunctional CD8+ T cells co-expressing PRF correlated negatively with HIV-1 DNA and CA usRNA (**Supplementary Figure 4**). Among the effector CD8+ T-cell percentages, the TNFα+ (r = -0.3366, p = 0.0168, insignificant after Bonferroni correction), and IFNγ+ (r = -0.4394, p = 0.0014, remains significant after Bonferroni correction) were negatively associated with HIV-1 CA usRNA (**Figure 1C**), whereas the CCL4+ CD8+ T-cell percentage was positively associated with HIV-1 CA usRNA (r = 0.5206, p = 0.0003, remains significant after Bonferroni correction; **Figure 1C**).

### CCL4- CCL5+ CD8+ T_EMRA_ Percentage Negatively Correlates With the HIV-1 Reservoir in ART Individuals

**Figure 2:**
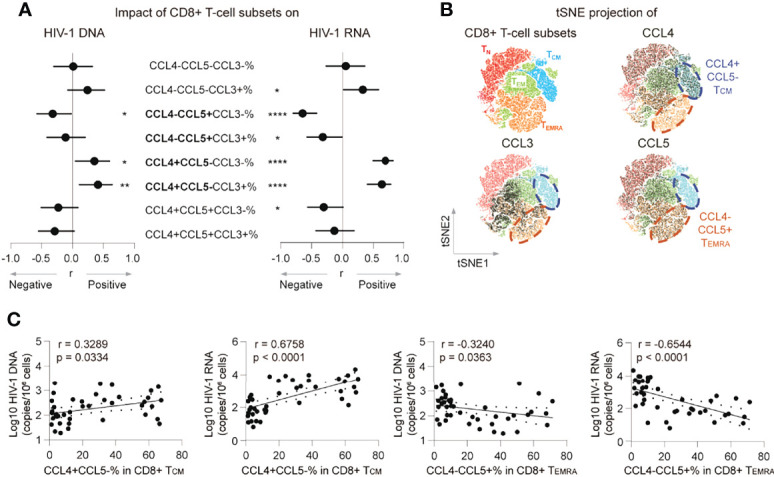
CCL4- CCL5+ CD8+ T_EMRA_ percentage negatively correlates with the HIV-1 reservoir in ART individuals. **(A)** Correlations of poly-functional CD8+ T cell percentages with CCL3, CCL4 and CCL5 secretion with HIV-1 viral reservoir size. **(B)** tSNE map of memory subset distribution (based on CD45RA, CD27, CCL3, CCL4 and CCL5) was present and indicated CD8+ T-cell subsets were plotted on tSNE map. Blue cycle highlighted CCL4+CCL5- T_CM_ subset, and yellow circle highlighted CCL4-CCL5+ T_EMRA_ subset. **(C)** Correlations of CCL4+CCL5-% in CD8+ T_CM_ or CCL4-CCL5+% in CD8+T_EMRA_ with HIV-1 DNA or CA usRNA levels. The correlations were evaluated using nonparametric Spearman correlation tests. Nonparametric Spearman’s r and p values are presented. *P < 0.05, **P < 0.01 and ****P < 0.0001.

Both CCL4 and CCL5 were reported as HIV-1-suppressive chemokines (15). A potential explanation for the opposite correlations between the HIV-1 reservoir and CCL4 and CCL5 producing CD8+ T cells is that these producing cells were distinctive subsets. To test the hypothesis and characterize their differences, we analyzed associations between CCL4 and CCL5 producing polyfunctional CD8+ T cells with HIV-1 reservoir, and CCL4 and CCL5 expression distribution across CD8+ T cells in t-SNE plots. The percentages of CCL4-CCL5+CCL3- CD8+ T cells negatively correlated with HIV-1 DNA (r = -0.3218, p = 0.0377, insignificant after Bonferroni correction) and CA usRNA (r = -0.6482, p < 0.0001, remains significant after Bonferroni correction), whereas CCL4+CCL5-CCL3- and CCL4+CCL5-CCL3+ CD8+ T-cell percentages positively correlated with HIV-1 DNA (r = 0.3542, p = 0.0214; r = 0.4111, p = 0.0068, respectively, neither significant after Bonferroni correction) and CA usRNA (r = 0.6987, p < 0.0001; r = 0.6347, p < 0.0001, respectively, both remain significant after Bonferroni correction) (**Figure 2A**; **Supplementary Figure 5**). In t-SNE plots, a T_CM_ subset is charactered with CCL4 producing but with rare CCL5 expression, whereas a T_EMRA_ subset is charactered with CCL5 producing but with rare CCL4 expression (**Figure 2B**). Further analysis showed, the percentage of CCL4+CCL5- in T_CM_ correlated positively with HIV-1 DNA (r = 0.3289, p = 0.0334) and CA usRNA (r = 0.6758, p < 0.0001), whereas and that of CCL4-CCL5+ in T_EMRA_ correlated negatively with HIV-1 DNA (r = -0.3240, p = 0.0363) and CA usRNA (r = -0.6544, p < 0.0001) (**Figure 2C**). These data indicated that CD8+ T_EMRA_ cells producing CCL5 and T_CM_ cells producing CCL4 restrained and facilitated HIV-1 reservoirs, respectively.

### T_VM_ Cells Are Enriched in CCL4- CCL5+ CD8+ T_EMRA_ Cells

**Figure 3:**
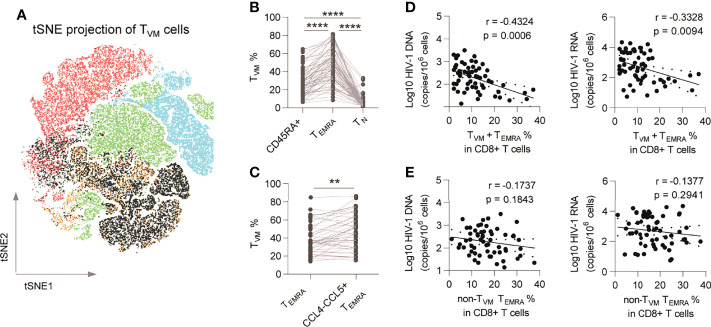
T_VM_ cells are enriched in CCL4- CCL5+ CD8+ T_EMRA_ and are functional subsets. **(A)** T_VM_ subset was plotted on tSNE map. **(B)** Comparison of T_VM_ subset percentages among CD45RA+ CD8+ T cells, CD8+ T_EMRA_ and CD8+ T_N_. Friedman test (for three group comparison) and Wilcoxon matched-pairs signed rank tests (for two group comparison) were performed, respectively. **(C)** Comparison of T_VM_ subset percentages in total CD8+ T_EMRA_ and CCL4-CCL5+ T_EMRA_. Wilcoxon matched-pairs signed rank tests was performed. **(D)** Correlations of T_VM_+ T_EMRA_ cell percentage in CD8+ T cells with HIV-1 DNA and CA usRNA levels. **(E)** Correlations of non-T_VM_ T_EMRA_ cell percentage in CD8+ T cells with HIV-1 DNA and CA usRNA levels. The correlations were evaluated using nonparametric Spearman correlation tests. **P < 0.01 and ****P < 0.0001.

We previously identified a restraining role of the T_VM_ subset in the HIV-1 reservoir (28) and here examined the connections of the T_VM_ subset with the HIV-1 reservoir and the potential effector mechanisms (**Supplementary Figure 2A** shows gating strategy for T_VM_ cell; **Supplementary Figure 2C** shows gating strategy for effector molecules of T_VM_ cells). Phenotypically, T_VM_ cells were mainly allocated to the CD8+ T_EMRA_ subpopulation (p < 0.0001 *vs.* CD45RA+ and CD8+ T_N_; **Figures 3A, B**). Furthermore, compared with total T_EMRA_, T_VM_ cells were enriched more in CCL4-CCL5+ T_EMRA_ (p = 0.0034; **Figure 3C**). Percentage of T_VM_+ T_EMRA_ in CD8+ T cells negatively correlated with HIV-1 DNA (r = -0.4324, p = 0.0006) and CA usRNA (r = -0.3328, p = 0.0094) (**Figure 3D**), whereas that of non-T_VM_ T_EMRA_ didn’t significantly correlate with HIV-1 DNA and CA usRNA (**Figure 3E**). These data support the notion that T_VM_ cells are the major functional anti-HIV-1 subset in CD8+ T_EMRA_ cells.

### T_VM_ Cells Are Transcriptionally Distinctive From CCL4+ T_CM_ Cells

**Figure 4:**
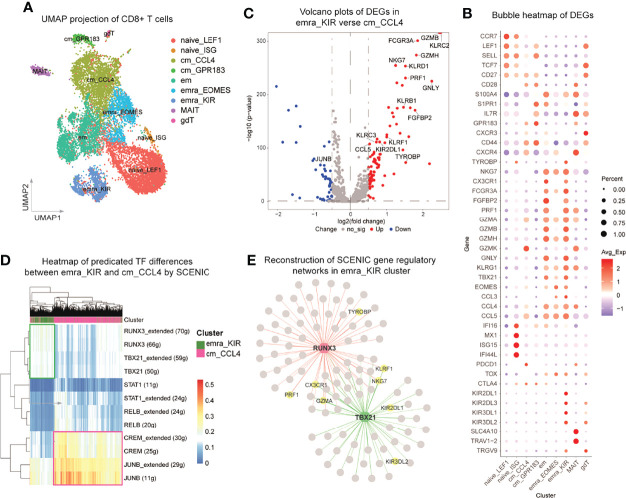
T_VM_ cells are transcriptionally distinctive from CCL4+ T_CM_ cells. scRNA-seq analysis of CD8+ T cells purified from three ART-treated individuals with viral suppression for more than 2 years. **(A)** Two-dimensional UMAP projection of 12033 cells by unsupervised clustering. **(B)** Bubble heatmap showing the gene expression distribution of selected canonical cell markers in the 9 clusters of CD8+ T cells. **(C)** Volcano plots showing the DEGs between emra_KIR and cm_CCL4. **(D)** Heatmap of predicated TF differences between emra_KIR and cm_CCL4 by SCENIC. **(E)** Reconstruction of SCENIC gene regulatory networks in emra_KIR cluster.

To further characterize the transcriptional profiling of T_VM_ cells, we analyzed the scRNA-seq datasets of purified CD8+ T cells from 3 ART-treated individuals (**Supplementary Figure 6A**). After normalization and batch correction, a total of 12033 CD8+ T cells were obtained. UMAP was applied to the transcriptional expression data, and identified 9 CD8+ T-cell clusters (**Figures 4A, B**; **Supplementary Table 1**). The major subsets of CD8+ T cells were naïve cells (*CCR7*+ *SELL*+ *CD27*+), cm cells (*CD28*+ *IL7R*+*GPR183*+), em cells (*CCR7*- *CD27*- *CXCR3*+), emra cells (*CCR7*- *CD27*- *GPR183*- *FGFBP2*+ *KLRG1*+), MAIT cells (*SLC4A10*+ *TRAV1-2*+) and gdT cells (*TRGV9*+). Two naïve CD8+ T-cell clusters were identified: naïve_LEF1 (*LEF1+*) and naïve_ISG (*IFI16+IFI44L+*). Two CD8+ cm clusters were identified: cm_GPR183 (*GPR183hi IL7R+ S1PR1+*) and cm_CCL4 (*CCL4+ PDCD1+ TOX+*). Two CD8+ emra clusters were identified: emra_EOMES (*EOMES+*) and emra_KIR (*KIR2DLs+ KIR3DLs+*). The proportions of CD8+ T-cell clusters in 3 individuals were presented in **Supplementary Figure 6C**.

Interestingly, the emra_KIR and cm_CCL4 subsets identified in scRNA-seq were characterized with robust expression of CCL5 and KIRs in T_EMRA_ and CCL4 in T_CM_, resembling T_VM_ and CCL4+ T_CM_ cells, respectively. The emra_KIR cells were also featured by high expression of cytotoxicity related genes, including *GZMB, GNLY* and *TBX21* (**Figure 4B**), which is in accordance with the phenotype at protein levels in T_VM_ cells as previously reported (28). GO analysis indicated that emra_KIR cluster was highly activated and characterized with upregulation of lymphocyte mediated immunity and T cell activation pathways, whereas cm_CCL4 cluster was potentially suppressive and characterized with downregulation of T cell activation pathways (**Supplementary Figure 6D**). To be specific, compared with cm_CCL4, emra_KIR up-regulated multiple effector molecules, such as *GZMB, PRF1, GZMH* and *CCL5*, supporting its superior antiviral function (**Figure 4C** and **Supplementary Table 2**). We further performed SCENIC analysis to identify potential TFs in emra_KIR and cm_CCL4 subsets. The results indicated that the responsible TFs in emra_KIR were RUNX3 and T-bet (encoded by *TBX21*) and those in cm_CCL4 were cAMP responsive element modulator (CREM) and JUNB (**Figures 4D, E** and **Supplementary Figure 6E**). RUNX3 and T-bet have been reported in regulating the function of CD8+ T cells and their expression are recognized as cardinal features of T_VM_ (28, 40, 41). In contrast, JUNB and CREM were proposed to exert suppressive functions in cm_CCL4, as indicated in CD4+ regulatory T cells (42, 43). Gene regulatory networks between TFs and DEGs in these two clusters were reconstructed (**Figure 4E**; **Supplementary Figure 6E** and **Supplementary Table 3**). RUNX3 and T-bet regulated the expression of several important effector molecules, such as *PRF1, CX3CR1* and *GZMA* (**Figure 4E**); whereas CREM and JUNB regulate gene expression maintaining T cell exhaustion, such as *PDCD1*, *BTG1* (44) and *ZFP36L2* (45) (**Supplementary Figure 6E**). These results supported the pro-activation roles of RUNX3 and T-bet in emra_KIR and suppressive function of CREM and JUNB in cm_CCL4.

### CCL5-Producing T_VM_ Cells are Negatively Correlated With HIV-1 Reservoir Size

**Figure 5:**
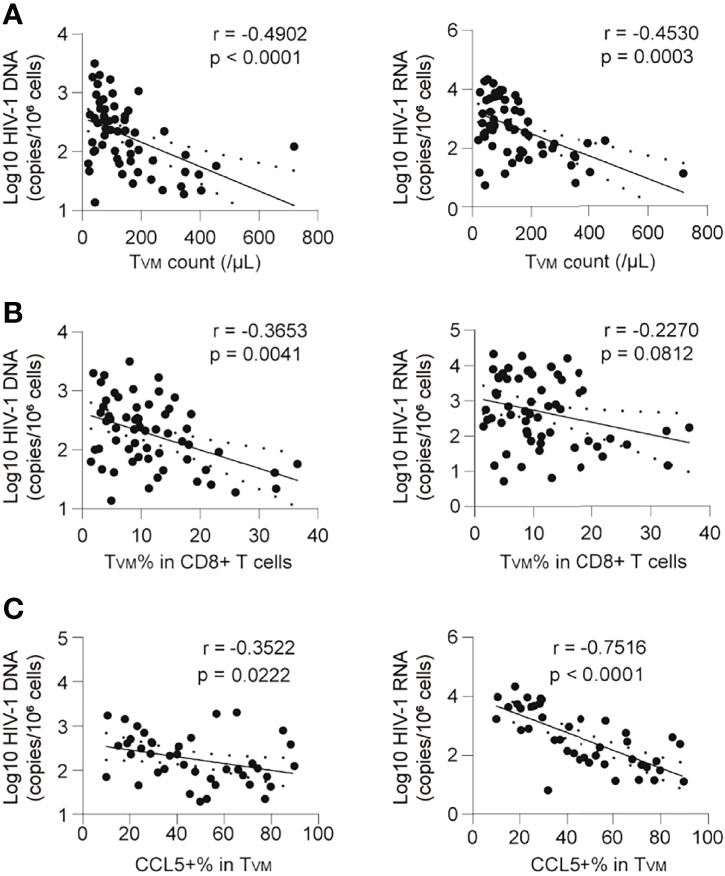
CCL5 produciing T_VM_ cells are negatively correlated with the HIV-1 reservoir in ART individuals. **(A)** Correlations of T_VM_ cell absolute count with HIV-1 DNA and CA usRNA levels. **(B)** Correlations of T_VM_ cell frequencies in CD8+ T cells with HIV-1 DNA and CA usRNA levels. **(C)** Correlations of CCL5 secretion in T_VM_ cells with HIV-1 DNA and CA usRNA levels. The correlations were evaluated using nonparametric Spearman correlation tests.

The frequency of T_VM_ correlated negatively with HIV-1 DNA and HIV-1 CA usRNA (**Figure 5**). The T_VM_ count (calculated according to its ratio with CD4+ T cells and the CD4 T cell count) and percentage in CD8+ T cells correlated negatively with HIV-1 DNA (r = -0.4902, p < 0.0001 and r = -0.3653, p = 0.0041, respectively; **Figures 5A, B**). The correlation between the T_VM_ count and HIV-1 CA usRNA was significantly negative (r = -0.4530, p = 0.0003), and the T_VM_ percentage in CD8+ T cells tended to negatively correlate with HIV-1 CA usRNA (r = -0.2270, p = 0.0812) (**Figures 5A, B**). In addition, total CCL5 producing T_VM_ percentage correlated negatively with HIV-1 DNA (r = -0.3522, p = 0.0222) and HIV-1 CA usRNA (r = -0.7316, p < 0.0001) (**Figure 5C**). Associations between CCL4 and CCL5 producing polyfunctional T_VM_ cells with HIV-1 reservoir were analyzed. CCL5 producing T_VM_ subset (including CCL4-CCL5+CCL3- and CCL4-CCL5+CCL3+) percentages negatively correlated with HIV-1 reservoir sizes, whereas CCL4 producing T_VM_ subset (including CCL4+CCL5-CCL3- and CCL4+CCL5-CCL3-) percentages positively correlated with HIV-1 reservoir sizes (**Supplementary Figure 7**). These data support the anti-HIV-1 roles of CCL5-secreting T_VM_ cells.

### T_VM_ Cells Restrained HIV-1 Reactivation *via* CCL5 Secretion

**Figure 6:**
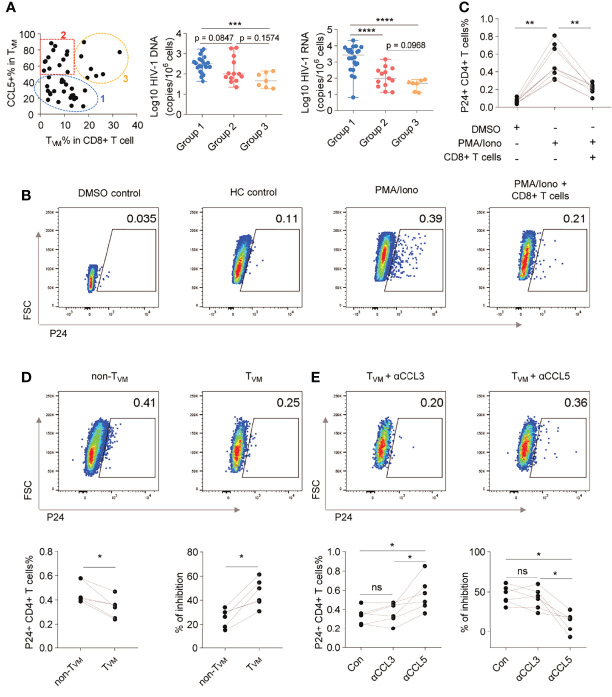
T_VM_ cells restrain HIV-1 reactivation *via* CCL5 secretion. **(A)** ART individuals were divided into 3 groups according to T_VM_ cell percentage in CD8+ T cells and CCL5 secretion in T_VM_ cells. HIV-1 reservoir levels were compared by using Kruskal-Wallis test (for three group comparison) and Mann Whitney test (for two group comparison). **(B)** Representative flow cytometry plots of HIV-1 P24 in CD4+ T cells. **(C)** Frequencies of HIV-1 P24+ cells in CD4+ T cells in the presence of autologous CD8+ T cells. **(D)** Representative gating, frequencies, and percentage inhibition of HIV-1 P24+ cells in CD4+ T cells after coculture with autologous T_VM_ or non-T_VM_ CD8+ T cells. **(E)** Representative gating, frequencies, and percentage inhibition of HIV-1 P24+ cells in CD4+ T cells after coculture with autologous T_VM_ cells with or without CCL3, or CCL5 blockade antibodies as indicated. Friedman test (for three group comparison) and Wilcoxon matched-pairs signed rank tests (for two group comparison) were performed. *P < 0.05, **P < 0.01, ***P < 0.001, ****P < 0.0001 and ns, not significantly.

Since both the frequency and the ability of CCL5 secretion in T_VM_ cells negatively correlated with HIV reservoir size, we further plotted every individual according to the T_VM_ percentage in CD8+ T cells and CCL5 production in T_VM_. As shown in **Figure 6A**, the individuals could be divided into 3 groups: Group 1 with low T_VM_ percentage and low CCL5 production; Group 2 with low T_VM_ percentage but high CCL5 production; Group 3 with high T_VM_ percentage and high CCL5 production. Compared with those in Group 1, the HIV-1 DNA size in Group 2 tended to be smaller (p = 0.0847), HIV-1 CA usRNA size in Group 2 was smaller (p < 0.0001) and these reservoir sizes were both significantly smaller in Group 3 (p = 0.0004, and p < 0.0001, respectively) (**Figure 6A**). No significant difference was found between Group 2 and Group 3 for HIV DNA, while Group 3 tended to have smaller CA usRNA size (p = 0.0968). This implies the crucial role of CCL5-secreting T_VM_ cells in restraining HIV reservoir.

To further confirm the viral inhibitory function of T_VM_ cells, we assayed viral inhibition *ex vivo* as described (32–34). The levels of HIV replication were evaluated by intracellular P24 staining using a single antibody (KC57 clone), which was easier to detect and positively correlated with a stricter HIV FLOW analysis (**Supplementary Figure 9**) (46). P24+ CD4+ T cells were significantly increased following PMA/Iono activation, but this condition was significantly suppressed by co-culture with CD8+ T cells (p < 0.05) (**Figures 6B, C**). Further results of viral inhibition assays showed that the T_VM_ subset suppressed HIV-1 replication more efficiently than the non-T_VM_ subset (p < 0.05; **Figure 6D**). The suppressive effects were partially reversed by αCCL5 (p < 0.05), but not by αCCL3 (**Figure 6E**).

## Discussion

This study explored the relationship between the HIV-1 reservoir and CD8+ T-cell subsets and functionality in PLWH undergoing long-term ART. Our study identified that CCL5-secreting virtual memory subset inversely correlated with HIV-1 reservoir size and might be utilized to shrink HIV-1 reservoir.

Given that the determinant roles of antigen-specific CD8+ T cells in controlling primary HIV-1 infection have been established, accumulating evidence suggested that the capability of HIV-1-specific CD8+ T cells is limited in eliminating latent cells carrying provirus, and shaping the viral epitope landscape in individuals undergoing ART (21–23). Alternatively, CD8+ T cells can exert anti-HIV-1 effects *via* a bystander mechanism (18, 20, 47, 48). The incapability of antigen-specific CD8+ T cells might be due to the escape mutation of HIV-1 epitopes (49), whereas viral killing mediated by bystander CD8+ T cells did not need MHC matching (18, 19). Our recent findings supported this notion. We found that T_VM_ cells TCR-independently and KIR-dependently restrain the HIV-1 DNA reservoir (28). KIRs are thought to function by releasing T_VM_ from functional restriction when target cells down-regulate MHC-I expression, like “miss-self” activation in NK cells. The inherent resistance of the reservoir to TCR-dependent antigen-specific recognition might be due to the downregulation of MHC-I by Nef expression following activation (50). However, such action of Nef would enable T_VM_ with killing capability. The present study further clarified that T_VM_ cells that were enriched in the classic T_EMRA_ subset inversely correlated with the size of the HIV-1 reservoir. We showed that T_VM_ cells restrained the HIV-1 reservoir more effectively than non-T_VM_ cells, which might be partially due to their abundant CCL5 secretion. Together, our findings suggest that T_VM_ cells could restrain latently infected cells in PLWH undergoing ART.

The association between CD8+ T-cell effector molecules and HIV-1 reservoirs has not been determined in detail. Prodger et al. (7) found that TNFα+ and IL-2+ CD8+ T cells are respectively positive and negative determinants of reservoir size measured by quantitative viral outgrowth assay (QVOA). In this study, we observed a significantly inverse correlation between IL-2+ CD8+ T cell percentage and HIV-1 reservoir sizes. Our polyfunctional analysis of CD8+ T cells revealed a positive correction between the frequency of TNFα+IFNγ-IL-2- CD8+ T cells and the size of the HIV-1 reservoir. Moreover, we did not observe a meaningful connection between IFNγ secretion by CD8+ T cells and HIV-1 reservoir size, which is consistent with the fact that the functionality of IFNγ secretion by CD8+ T cells does not correlate with HIV-1 controls (51, 52).

For beta chemokines, we consistently found that CCL5 was negative determinants of reservoir size in both CD8+ T cells and T_VM_ cells. In sharp contrast to CCL3 and CCL5, CCL4+ CD8+ T cells correlated positively with HIV-1 CA usRNA, indicating heterogeneity among CD8+ T cells that secrete β-chemokines. Accordingly, distinctive CCL4 and CCL5-producing CD8+ T cell subsets with different regulators were identified in primary human T cells by genome-wide CRISPR screening (53). Indeed, our flow cytometry detection and scRNA-seq analysis identified a unique CCL4+CCL5- subpopulation within the classic T_CM_ subset, the frequency of which correlated positively with HIV-1 reservoir size. Similarly, CD8+ T cells that produce CCL4 are expanded in individuals with a poor immune reconstitution who undergo ART (16). A recent study on melanoma found CCL4-expressing CD8+ T cells were robust expressors of Lag-3, which supports the notion that this subset might be exhausted (54). Using scRNA-seq data, we performed SCENIC analysis and identified CREM and JUNB as potential TFs in cm_CCL4. Further studies are needed to precisely characterize the phenotypes and functions of CCL4+ CD8+ T cells, especially in chronic viral infection. Moreover, in the *ex vivo* assay, we observed that CCL5 blockade diminished the virus-inhibitory effect of T_VM_ cells. Since ART was not included in the assay, CCL5 might be multi-functional in HIV inhibition, either by preventing of transmission or by directly inhibiting of viral replication. The latter could be possibly explained by CCL5 induced activation of signal transduction cascades through its receptors, such as CCR5, CCR1 and CCR3 (55).

Our study has several limitations. We measured the HIV-1 reservoir as HIV-1 DNA and CA usRNA, but not as being replication-competent by QVOA or as intact provirus by full-length sequencing or intact proviral DNA assays. Despite these indicators were highly correlated (56), the characteristics of latent reservoirs cannot be fully determined by HIV-1 DNA or CA usRNA. The HIV-1 reservoir was not measured at multiple timepoints. Thus, the dynamics and decay of the HIV-1 reservoir could not be determined. We also did not exclude HIV-1-specific CD8+ T cells from the analyzed CD8+ T cells. Nevertheless, our findings indicated that CCL5-secreting T_VM_ cells could limit the size of the HIV-1 reservoir, which might be helpful to design CD8+ T cell-based therapeutic strategies for the cure of the disease.

## Data Availability Statement

The datasets presented in this study can be found in online repositories. The names of the repository/repositories and accession number(s) can be found in the article/**Supplementary Material**.

## Ethics Statement

The studies involving human participants were reviewed and approved by the Fifth Medical Center of the Chinese PLA General Hospital and the Fourth People’s Hospital of Nanning. The patients/participants provided their written informed consent to participate in this study.

## Author Contributions

F-SW, and CZhang conceived the study, supervised the work performed, wrote the manuscript, and constructed the figures with WH, Y-JL, CZhen, and Y-YW. The participants were enrolled by H-HH, JZ and Y-QZ. The PBMC samples were collected and isolated by G-CH, S-RM, Y-QQ, and F-YW. Clinical data were collected by WH, Y-YW, and J-HJ. Flow cytometry experiments were performed by WH, Y-YW, JL, M-JZ, Y-LF, X-YL, and X-HY, with technical support of C-BZ, and J-HY. Experiment to quantify viral RNA was performed by TY and X-WW. scRNA-seq data was analyzed by CZhen and PZ. J-WS, XF, Y-MJ, R-NX, J-YZ, C-BZ, and LH edited the manuscript and provided comments and feedback. F-SW, and MS managed the study team and oversaw data analysis. All authors read and approved the final manuscript.

## Funding

This study was supported by Innovation Groups of the National Natural Science Foundation of China (grant no. 81721002), the National Science and Technology Major Project (grant no. 2018ZX10302104-002), National Natural Science Foundation of China (grant nos. 81901617 and 82101837) and the Beijing Natural Science Foundation (grant no. 7222171).

## Conflict of Interest

The authors declare that the research was conducted in the absence of any commercial or financial relationships that could be construed as a potential conflict of interest.

## Publisher’s Note

All claims expressed in this article are solely those of the authors and do not necessarily represent those of their affiliated organizations, or those of the publisher, the editors and the reviewers. Any product that may be evaluated in this article, or claim that may be made by its manufacturer, is not guaranteed or endorsed by the publisher.
