# Supplementary material for: CCL5-Secreting Virtual Memory CD8+ T Cells Inversely Associate With Viral Reservoir Size in HIV‐1−Infected Individuals on Antiretroviral Therapy
Source: Front Immunol. 2022 May 26;13:897569. doi: 10.3389/fimmu.2022.897569 (PMC9204588; doi:10.3389/fimmu.2022.897569)
Supplement: Supplementary file 4 [file DataSheet_4.pdf]

SUPPLEMENTARY MATERIALS

CCL5-secreting virtual memory CD8+ T cells inversely associate with viral reservoir size in HIV-1−infected individuals on antiretroviral therapy

Wei H et al.

Supplementary Figure 1. Correlations of clinical parameters with HIV-1 DNA or CA usRNA levels.

Supplementary Figure 2. Representative flow cytometry plot.

Supplementary Figure 3. Correlations of CD8+ T_{CM}, T_{EMRA} and functional CD8+ T cell percentages with HIV-1 viral reservoir size.

Supplementary Figure 4. Correlations of poly-functional CD8+ T cell percentages with HIV-1 viral reservoir size.

Supplementary Figure 5. Correlations of CCL4+CCL5- and CCL4-CCL5+ CD8+ T cell percentages with HIV-1 viral reservoir size.

Supplementary Figure 6. scRNA-seq analysis of CD8+ T cells.

Supplementary Figure 7. Correlations of poly-functional TVM cell percentages with HIV-1 viral reservoir size.

Supplementary Figure 8. Correlations of ART duration with HIV-1 viral reservoir size and immune subset parameters.

Supplementary Figure 9. Correlations of P24+ percentages between HIV FLOW and a single antibody analysis in seven HIV-1−infected patients.

Supplementary Table 1. Canonical cell marker expression in 9 clusters identified in 3 ART-treated individuals by scRNA-seq analysis. We generated bubble heatmap of canonical cell marker expression in the 9 clusters of CD8+ T cells (**Figure 3E**).

Supplementary Table 2. DEGs in emra_KIR cluster verse cm_CCL4 cluster. We generated volcano plots and gene enrichment analyses to show the DEGs in emra_KIR cluster verse cm_CCL4 cluster (**Figure 3F** and **Supplementary Fig. 6D**).

Supplementary Table 3. Predicted transcription factors and their targets in emra_KIR and cm_CCL4 clusters. We generated heatmap of predicated transcriptional factor differences and gene regulatory networks in emra_KIR and cm_CCL4 clusters by SCENIC analysis (**Figure 3 G, H** and **Supplementary Fig. 6E**). This table comprises the predicted transcription factors and their targets.

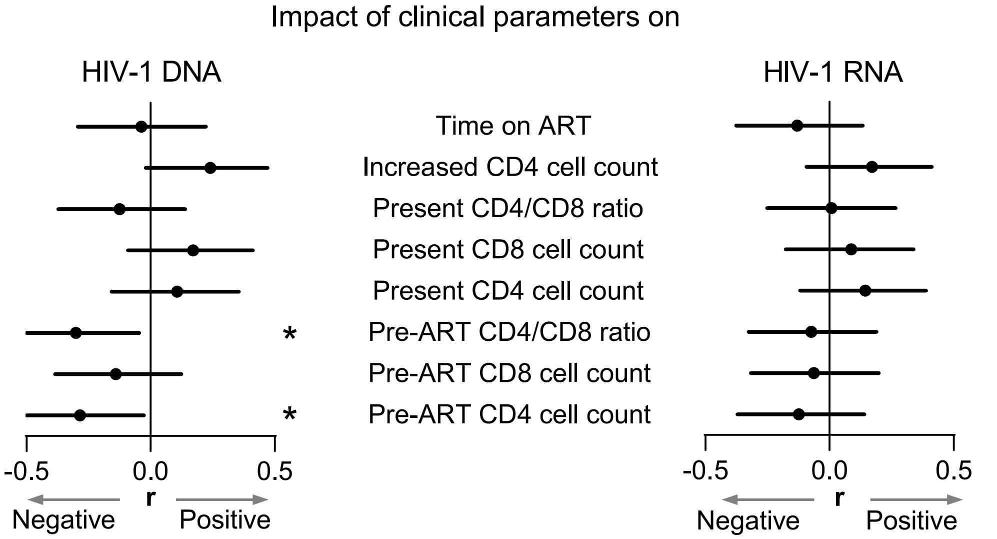

Supplementary Figure 1. Correlations of clinical parameters with HIV-1 DNA or CA usRNA levels. Correlations were evaluated using nonparametric Spearman correlation tests. Black dots denote nonparametric Spearman r, and black lines denote 95% confidence interval. $*P < 0.05$.

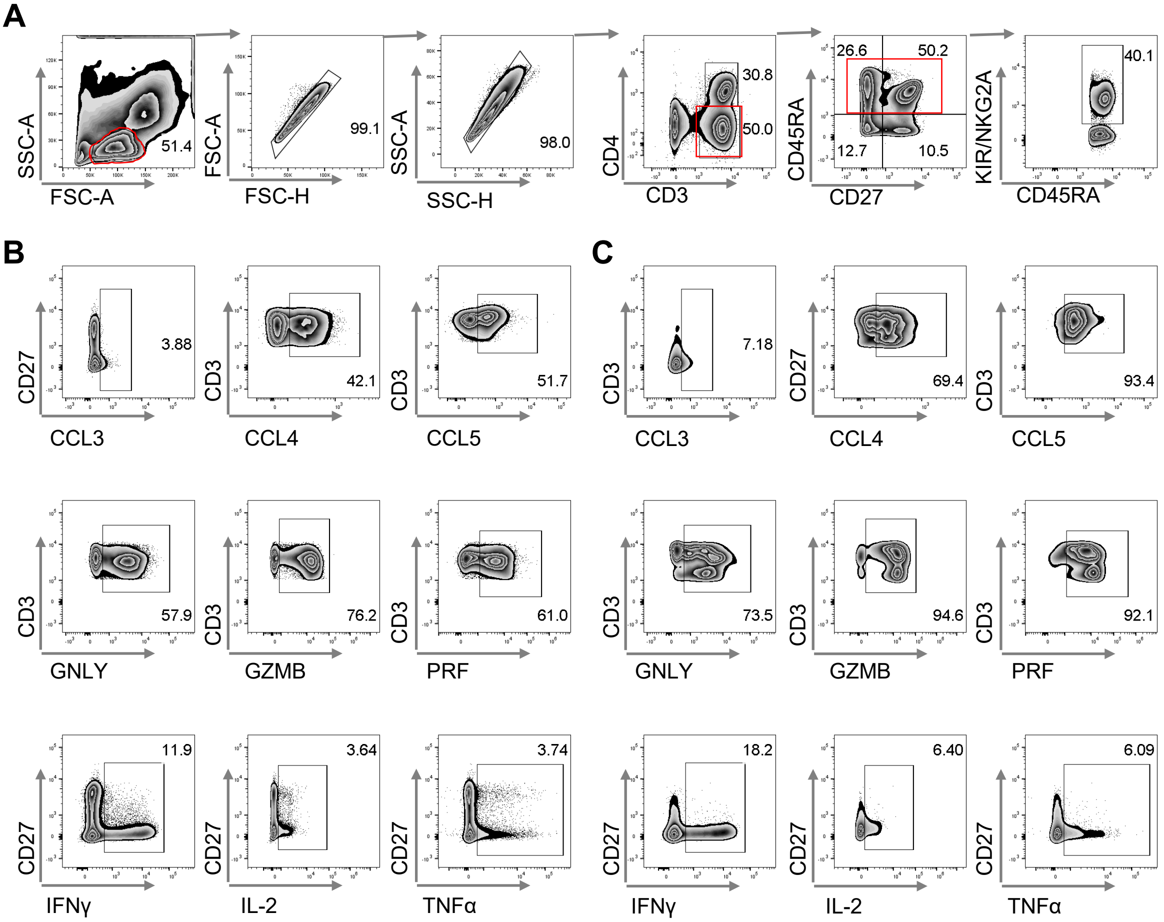

Supplementary Figure 2. Representative flow cytometry plots. (A) Gating strategies for analysis of CD4+ and CD8+ T cell subsets based on CD45RA and CD27, and T_{VM} based on CD8, CD45RA and pan-KIR/NKG2A. (B) Representative flow cytometry plots of indicated effector molecules' expression in CD8+ T cells. (C) Representative flow cytometry plots of indicated effector molecules' expression in T_{VM} cells.

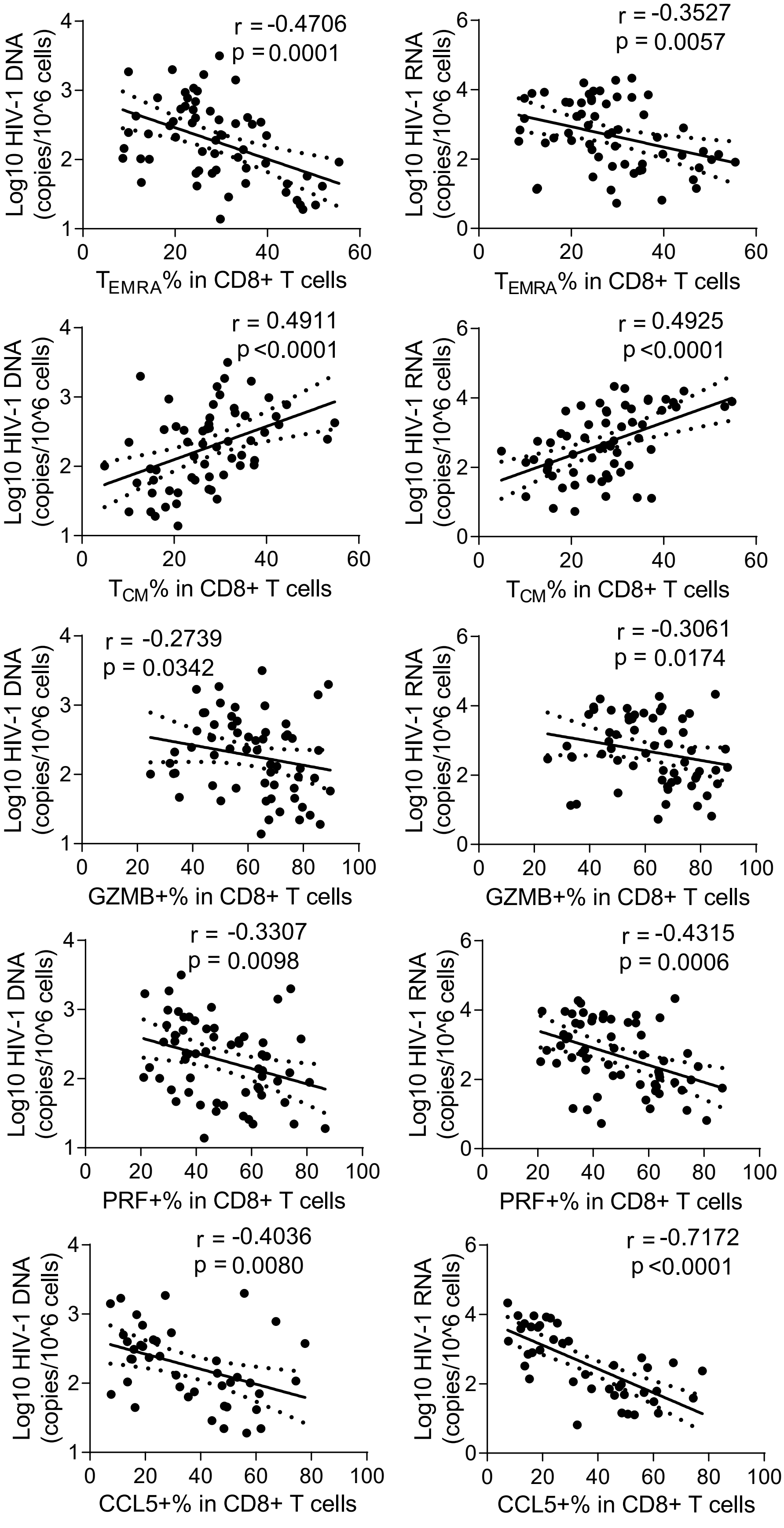

Supplementary Figure 3. Correlations of CD8+ T_{CM}, T_{EMRA} and functional CD8+ T cell percentages with HIV-1 viral reservoir size. Correlations were evaluated using nonparametric Spearman correlation tests. Nonparametric Spearman's r and p values are presented.

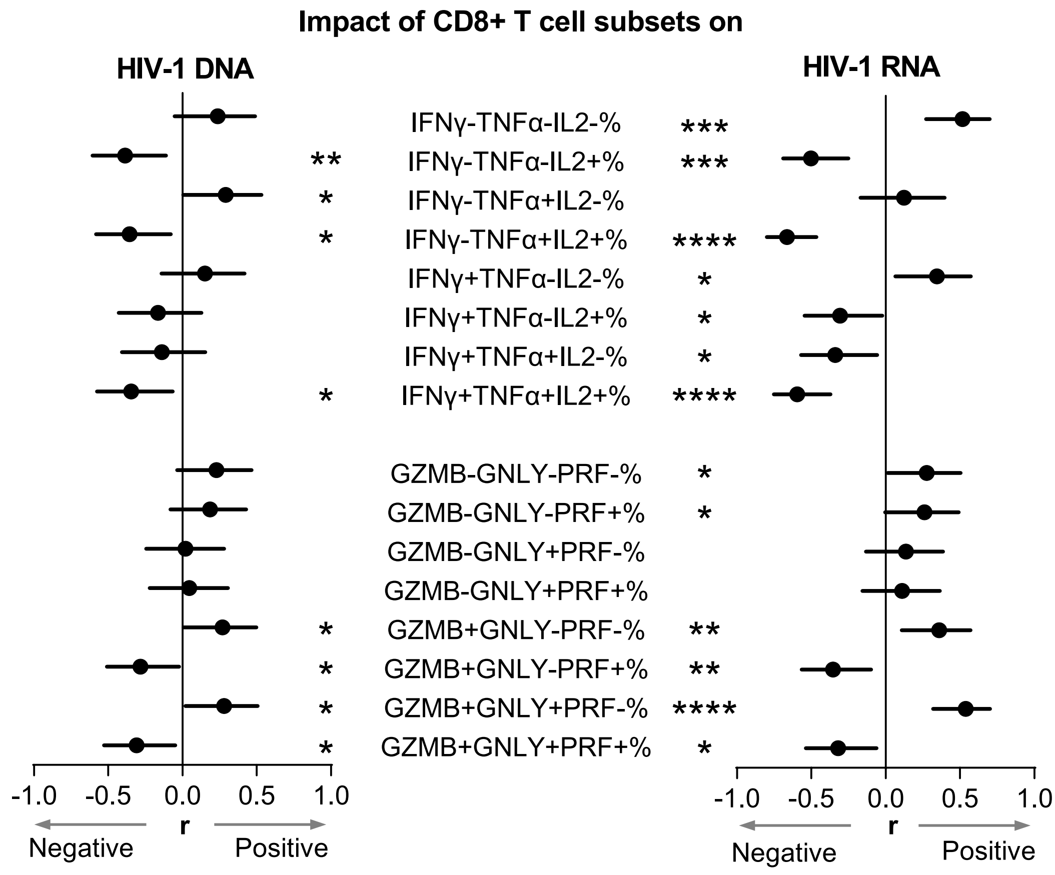

Supplementary Figure 4. Correlations of poly-functional CD8+ T cell percentages with HIV-1 viral reservoir size. Correlations were evaluated using nonparametric Spearman correlation tests. Black dots denote nonparametric Spearman r, and black lines denote 95% confidence interval. *P < 0.05, **P < 0.01, ***P < 0.001, ****P < 0.0001 respectively.

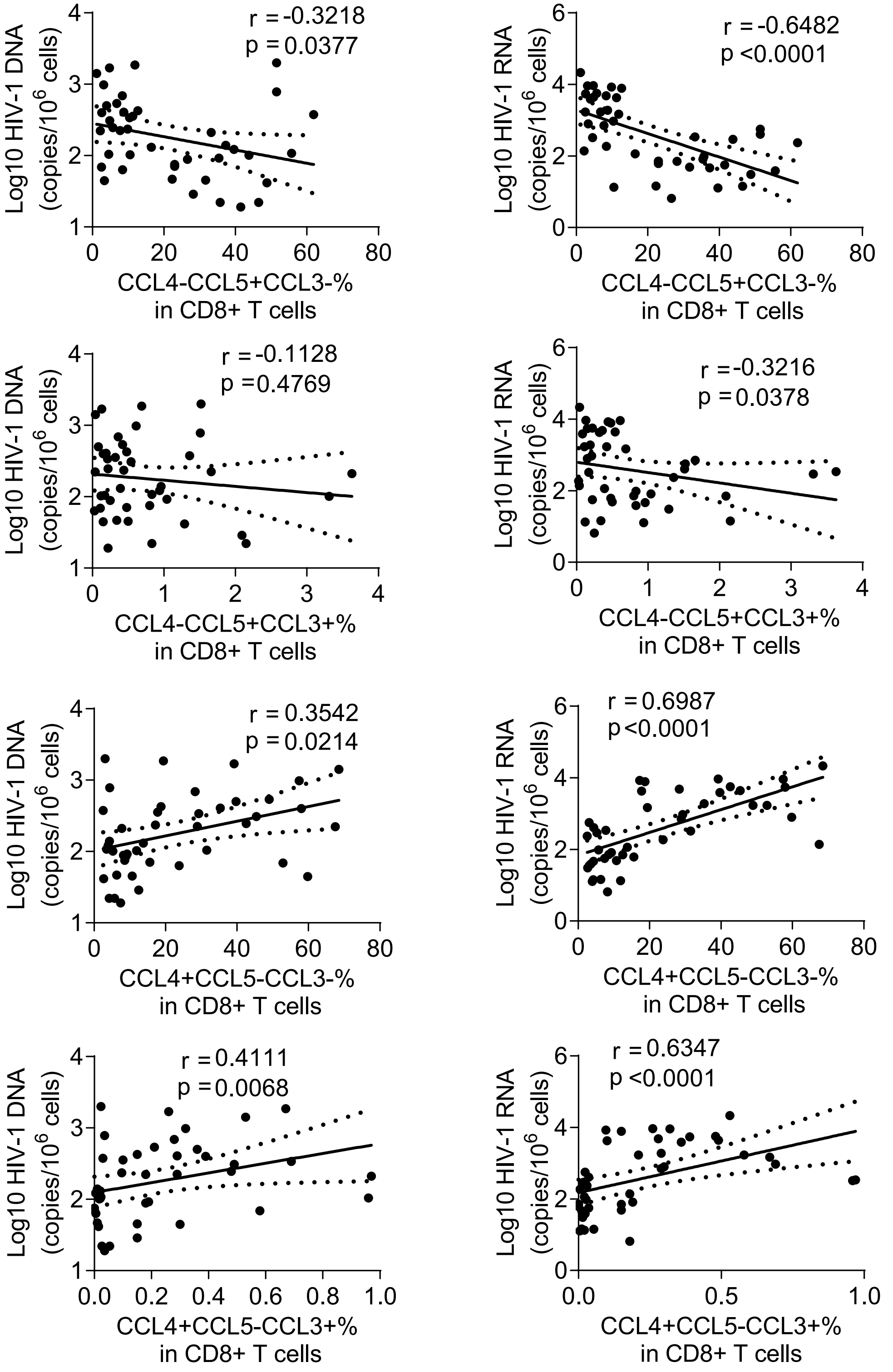

Supplementary Figure 5. Correlations of CCL4+CCL5- and CCL4-CCL5+ CD8+ T cell percentages with HIV-1 viral reservoir size. Correlations were evaluated using nonparametric Spearman correlation tests. Nonparametric Spearman's r and p values are presented.

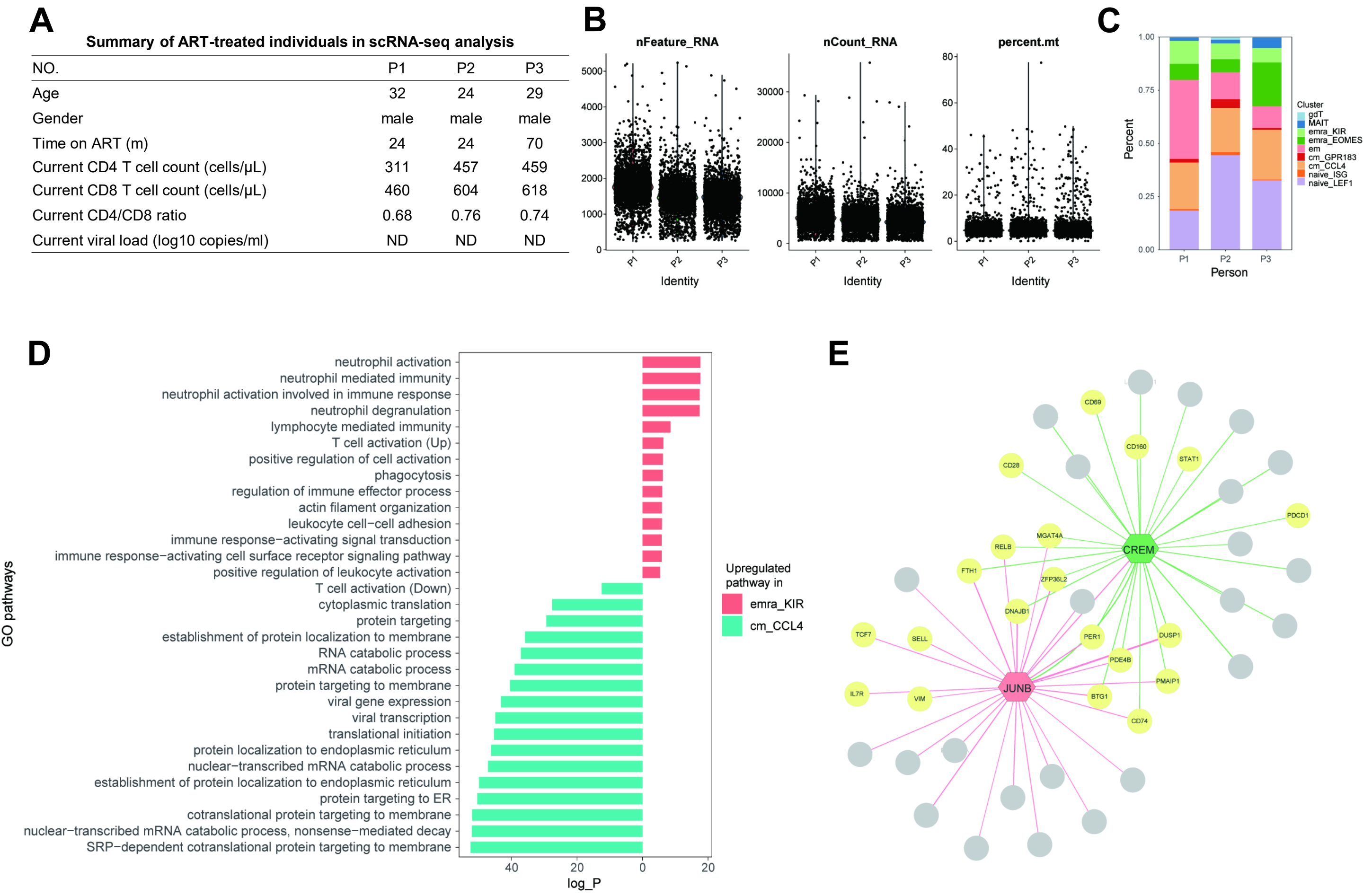

Supplementary Figure 6. scRNA-seq analysis of CD8+ T cells. (A) Characteristics of 3 ART-treated individuals with scRNA-seq data. (B) Number of feature read counts (left), number of read counts (middle), and percentage of mitochondrial genes (right) in each sample. (C) The proportions of different CD8+ T cell clusters in everyone. (D) Gene ontology analyses of the DEGs dominated in emra_KIR and cm_CCL4 clusters subset. (E) Reconstruction of SCENIC gene regulatory networks in cm_CCL4 cluster analyzed by SCENIC.

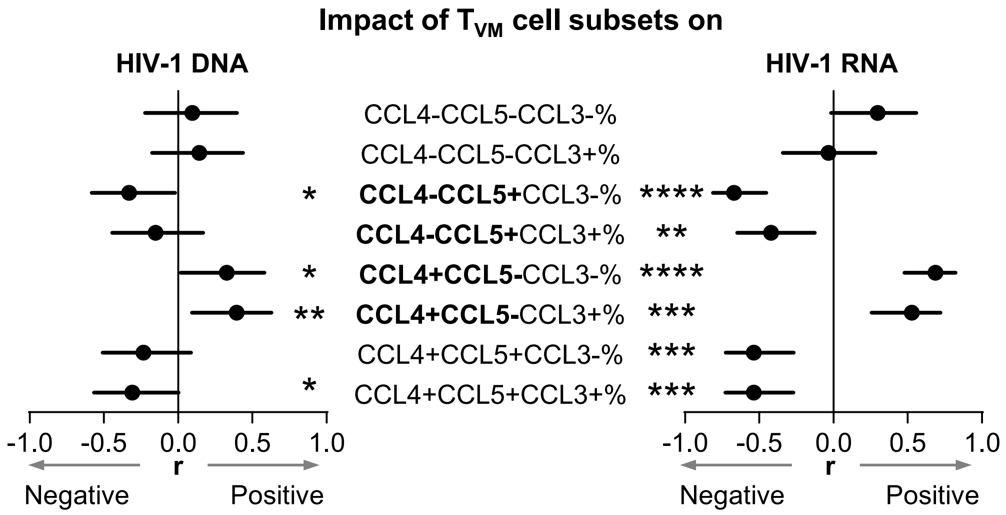

Supplementary Figure 7. Correlations of poly-functional T_{VM} cell percentages with HIV-1 viral reservoir size. Correlations were evaluated using nonparametric Spearman correlation tests. Black dots denote nonparametric Spearman r, and black lines denote 95% confidence interval. $*P < 0.05$, $**P < 0.01$, $***P < 0.001$, $****P < 0.0001$ respectively.

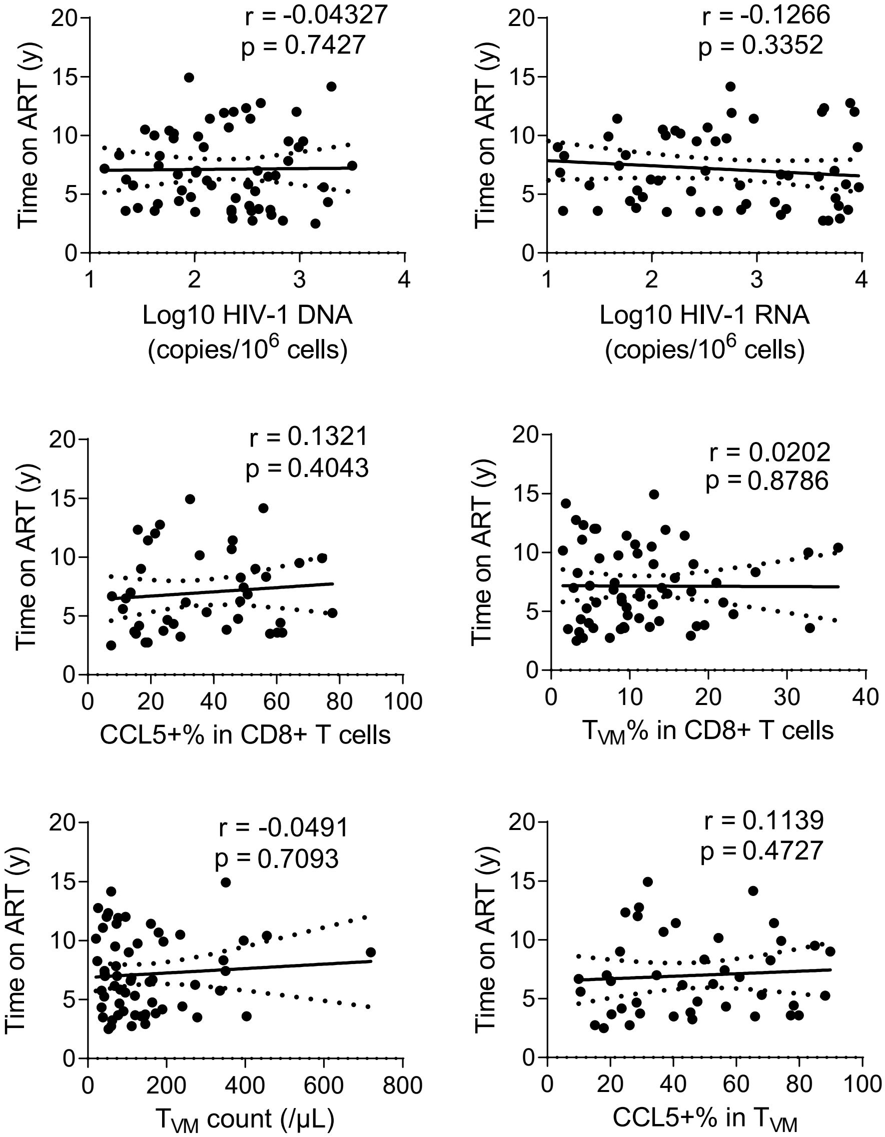

Supplementary Figure 8. Correlations of ART duration with HIV-1 viral reservoir size and immune subset parameters. Correlations were evaluated using nonparametric Spearman correlation tests. Nonparametric Spearman's r and p values are presented.

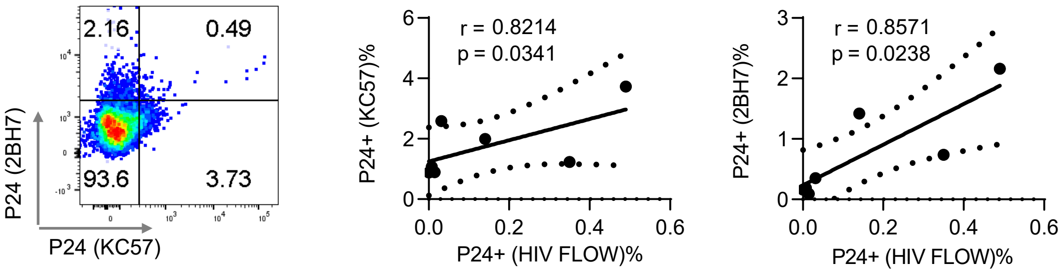

Supplementary Figure 9. Correlations of P24+ percentages between HIV FLOW and a single antibody analysis in seven HIV-1−infected patients. Representative flow cytometry plots for P24 gating using HIV FLOW analysis. Correlations were evaluated using nonparametric Spearman correlation tests. Nonparametric Spearman's r and p values are presented.
